# The regulation of NONO by USP11 via deubiquitination is linked to the proliferation of melanoma cells

**DOI:** 10.1111/jcmm.16243

**Published:** 2020-12-25

**Authors:** Peifu Feng, Ling Li, Jing Dai, Lingli Zhou, Jing Liu, Jinfeng Zhao, Xiaodong Li, Neng Ling, Siyuan Qiu, Lin Zhang, Tiantian Xie, Yinglei Chen, Michael J. Donovan, Tianhuan Peng, Jianhui Song, Mao Ye

**Affiliations:** ^1^ Molecular Science and Biomedicine Laboratory State Key Laboratory for Chemo/Biosensing and Chemometrics College of Biology College of Chemistry and Chemical Engineering Aptamer Engineering Center of Hunan Province Hunan University Changsha China; ^2^ Molecular Biology Research Center & Center for Medical Genetics School of Life Sciences Central South University Changsha China; ^3^ Key Laboratory of Nanobiological Technology of Chinese Ministry of Health Xiangya Hospital Central South University Changsha China; ^4^ Department of Pediatrics Xiangya Hospital Central South University Changsha China

**Keywords:** deubiquitination, melanoma, NONO, proliferation, USP11

## Abstract

Ubiquitin‐specific protease 11 (USP11) has been implicated in the regulation of DNA repair, apoptosis, signal transduction and cell cycle. It belongs to a USP subfamily of deubiquitinases. Although previous research has shown that USP11 overexpression is frequently found in melanoma and is correlated with a poor prognosis, the potential molecular mechanism of USP11 in melanoma remains indefinitive. Here, we report that USP11 and NONO colocalize and interact with each other in the nucleus of melanoma cells. As a result, the knockdown of USP11 decreases NONO levels. Whereas, overexpression of USP11 increases NONO levels in a dose‐dependent manner. Furthermore, we reveal that USP11 protects NONO protein from proteasome‐mediated degradation by removing poly‐ubiquitin chains conjugated onto NONO. Functionally, USP11 mediated melanoma cell proliferation via the regulation of NONO levels because ablation of USP11 inhibits the proliferation which could be rescued by ectopic expression of NONO protein. Moreover, a significant positive correlation between USP11 and NONO concentrations was found in clinical melanoma samples. Collectively, these results demonstrate that USP11 is a new deubiquitinase of NONO and that the signalling axis of USP11‐NONO is significantly involved in melanoma proliferation.

## 1 INTRODUCTION

Melanoma is a malignant tumour derived from cutaneous melanocytes.^1^ In 2018, it was estimated that 287 723 patients were diagnosed, and 60,712 patients died from this cancer worldwide.^2^ In the past decade, clinical treatment of melanoma has progressed dramatically, especially after immunotherapies based on CTLA‐4 (cytotoxic T‐lymphocye‐associated protein 4) or PD‐1/PD‐L1 (programmed death protein 1 / programmed death‐ligand 1) were used in clincial treatment.^3^ However, the five‐year survival rate of progressive metastatic melanoma remains at a modest level of 22.5%. Therefore, a deeper understanding of the molecular mechanisms in melanoma is needed in order to spur development of novel therapeutic strategies for this malignancy.

The ubiquitin‐proteasome system (UPS) has a substantial role in the degradation of targeted protein, which is responsible for the degradation of about 80%‐90% of cellular normal and abnormal proteins.^4^ This thereby influences various cell activities such as cell proliferation, apoptosis, cell cycle and signal transduction. Its malfunction may result in a multitude of human pathologies including cancer.^5^ Similar to other post‐translational regulations, the reverse reaction of ubiquitination is termed deubiquitination by which ubiquitin is cleaved from substrate proteins by peptidases called deubiquitinating enzymes (DUBs). In humans, there are more than 100 kinds of DUBs that can be classified into 7 evolutionarily conserved subfamilies. The two newest members MINDY and ZUP1 were discovered relatively recently.^6^ Ubiquitin‐specific protease 11 (USP11) is a member of the USP subfamily of DUBs, which has been implicated in the regulation of various cellular functions by controlling its substrates’ stability.^7^ Likewise, USP11 malfunction has been found in many types of cancer and related in tumour development and progression.^8^, ^9^, ^10^ In melanoma, overexpression of USP11 has been frequently observed and is correlated with poor prognosis.^11^

The NONO protein, also referred to as 54 kD Nuclear RNA‐ and DNA‐binding protein (p54nrb), is a member of the DBHS family, whose involvement can be seen at virtually every step of the gene regulation process.^12^, ^13^ Meanwhile, the NONO protein is also involved in many important biological pathways, such as the cyclic AMP pathway,^14^ NF‐κB signalling pathway,^15^ Akt and Erk1/2 signalling pathways.^16^ To date, NONO has been found to be associated with cancers as either an oncogene or tumour suppressor.^17^ NONO is generally downregulated in ER (estrogen receptor)‐negative breast cancer.^18^ By contrast, growing evidence indicates that NONO is overexpressed in a multitude of cancers, such as bladder cancer,^19^ lung cancer,^20^, ^21^ esophageal squamous cell carcinomas (ESCC) ^16^ and prostate cancer.^22^, ^23^ Moreover, NONO protein level can be used as an independent prognostic factor for a number of cancers^24^, ^25^, ^26^, ^27^ including melanoma.^28^

Previous studies demonstrated that not all melanoma/melanocyte cell lines have a definitive correlation between NONO mRNA expression and their protein levels.^28^, ^29^, ^30^ This implies additional post‐transcriptional mechanisms including ubiquitination may be involved in the regulation of NONO protein levels. The phosphorothioate oligonucleotides were reported to cause the degradation of NONO protein in a proteasome‐dependent manner.^31^ Most recently, two ubiquitin ligases (E3) were found to be involved in the degradation.^29^, ^30^ However, further research is needed to determine whether ubiquitinated NONO can be recycled.

We identified USP11 as the first deubiquitinase of NONO protein in this study. USP11 interacted with NONO and reversed its poly‐ubiquitination. As a result, USP11 positively regulated NONO levels by protecting it from ubiquitin‐dependent degradation. We also demonstrated that USP11 promoted the melanoma cells proliferation and tumorigenesis via NONO. Moreover, a significant positive correlation between USP11 and NONO concentrations was found in clinical melanoma samples, implying that USP11 is a potential target candidate in the diagnosis and treatment of melanoma.

## 2 MATERIALS AND METHODS

### 2.1 Cell culture and reagents

SK‐Mel‐28, A375 and HEK293 cells were kindly provided from Cell Bank, Chinese Academy of Sciences. All cells were cultured using DMEM (Gibco) with 10% fetal bovine serum (FBS) in a cell incubator under standard conditions at 37°C and 5% CO_2_. Reagents used are as follows: GST‐tag Purification Resin (Beyotime Biotechnology, Cat. P2250), GSH (Beyotime Biotechnology, Cat. S0073), protease inhibitor cocktail (Bimake, Cat. B14012), LipoMax plasmid transfection reagent (SUDGEN, Cat.17052012), GenMute siRNA transfection reagent (SignaGen Laboratories, Cat. SL100568), MG132 (Sigma, Cat. C2211), M‐PER™ Mammalian Protein Extraction Reagent (Thermo Fisher, Cat.78501), Protein G Magnetic Beads (Thermo Fisher, Cat. 10004D), Clean‐blot™ IP detection reagent (HRP) (Thermo Fisher, Cat. 21230), RIPA Lysis Buffer (Beyotime, Cat.P0013B) and Cell Counting Kit‐8 (CCK‐8) (Bimake, Cat. B34304).

### 2.2 Plasmids

A 3 × Flag‐tagged USP11 expression plasmid was constructed by cloning the entire length of USP11(wild‐type USP11, WT USP11) into pCMV‐Tag2B (with a 3 × Flag tag). A 3 × Myc‐tagged NONO expression plasmid was generated by cloning the entire length of NONO into pcDNA3.1 (with a 3 × Myc tag). Tail truncations and catalytically inactive mutants (C275S/C283S) of USP11 were cloned into the pCMV‐Tag2B (with a 3 × Flag tag). A GST‐tagged *E.coli* expression plasmid was generated by inserting the full length of USP11 into the pGEX‐4T‐1 vector.

### 2.3 Antibodies

Anti‐USP11 (catalog no. sc‐365528), anti‐Ub (catalog no. sc‐166553) and anti‐NONO (catalog no. sc‐376865) antibodies were purchased from Santa Cruz. Anti‐USP11 (EPR4346) and anti‐Myc (2276S) antibodies were provided by Abcam. Three antibodies against NONO were obtained from Sangon Biotech (catalog no. D199144), MBL Life science (catalog no. RN092PW), Proteintech Group, Inc (catalog no. 11058‐1‐AP). Anti‐Flag (catalog no. M185‐3L), anti‐HA (catalog no. M180‐3) and anti‐Myc (catalog no.M192‐3) were purchased from Medical & Biological Laboratories. Anti‐GAPDH (catalog no. KC‐5G4) antibody was bought from Kangchen Biotech.

### 2.4 CCK‐8 assay

The stable cell lines for low expression of USP11 were generated using specific lentiviral short hairpin RNAs (shRNAs) into SK‐Mel‐28 and A375, then NONO or empty vector was introduced into the stable cells for 24 hours. Specified cells were seeded in quadruplicate into the wells of 96‐plate at a specified density of 8 × 10^3^ cells per well. CCK‐8 assays were performed according to the manufacturer's protocols for four consecutive days in triplicate.

### 2.5 Colony formation assay

The USP11 lower‐expression A375 stable cell lines were transfected with NONO‐pcDNA3.1 constructs or relative empty plasmid for 24 hours and then plated in 6‐well plates at an optimized density of 8000 cells/well, respectively. The cells were maintained in selective medium containing DMEM and 10% FBS and 800 ug/mL G418 (Sigma‐Aldrich). After 2 weeks, cells were fixed in 4% paraformaldehyde for 30 minutes. Using 0.1% crystal violet, the cells were then stained. Colony numbers present were subsequently counted.

### 2.6 Immunohistochemistry (IHC)

The tissue arrays of melanoma/adjacent tissue of melanoma/normal skin tissue samples were purchased from Alenabio (ME803a). The arrays were incubated in Anti‐USP11(Santa Cruz, 1:100) or anti‐NONO (Sangon Biotech, 1:100) at room temperature overnight, rinsed twice with DPBS (Gibco), and incubated with a secondary antibody. After staining, arrays were scanned with the use of a Pannoramic^®^ MIDI digital slide scanner (3DHISTECH).

IHC scores of USP11 and NONO were assessed by two independent practising pathologists. The scores were quantified based upon a 4‐point system to rate the intensity of cytoplasm and nuclear staining. The scoring system ranged from 0 to 3 for none, light, medium or dark staining, respectively. Finally, the groups were classified as low expression (0 and 1) or high expression (2 and 3). Statistical analysis of the correlation between USP11 and NONO was calculated based upon the *χ* ^2^ test.

### 2.7 Immunofluorescence Staining

Cells were cultured for 24 hours in Lab‐Tek chambers. PBS was used to wash them (10 min × 3). They were then fixed with 4% paraformaldehyde for 15 minutes, washed with PBS again (10 min × 3), permeabilized in 0.2% Triton X‐100 for 10 minutes, blocked in 5% BSA for 1 hour, and then incubated overnight at 4°C with the designated primary antibodies. The antibodies against USP11 (1:200) and NONO (1:200) antibodies were utilized to monitor endogenous protein expression. Secondary antibodies conjugated to DyLight Fluorescent Dyes (ImmunoReagents, catalog no. GtxRb‐003‐D488NHSX; catalog no. GtxMu‐003‐D594NHSX; catalog no. GtxRb‐003‐D594NHSX; catalog no. GtxMu‐003‐F488NHSX) were used to label proteins for 1 hour at room temperature, followed by 3 washing cycles with PBS. At last, DAPI was used to counterstain the cells at room temperature for 5 minutes. This was performed in order to visualize nuclear DNA. A confocal microscope (Zeiss LSM510) was used to develop the fluorescence images.

### 2.8 Western Blotting and Immunoprecipitation

Cell samples were lysed with M‐per or RIPA lysis buffer supplemented with a protease inhibitor cocktail for 15 minutes on ice, followed by a 15 000 *g* centrifugation for 10 minutes. Supernatants were collected. Total protein concentrations were determined with the NanoDrop (ThermoFisher, NanoDrop 2000/2000c UV‐Vis or NanoDrop Lite UV). Lysates were resolved on 8%, 10% or 12% gels using SDS/PAGE according to the molecular weights of the targets and then transferred to PVDF membranes for Western blotting. Protein bands were visualized using ECL detection reagents (Advansta catalog no. K‐12045‐D50). For immunoprecipitation, equal amounts of lysate (total proteins) were incubated overnight at 4°C with 3 μg of either a relevant primary antibody or an isotype‐matched normal IgG. Each sample was incubated for an additional hour with 30 μL of Protein G Magnetic Beads. The beads subsequently underwent three wash cycles with coimmunoprecipitation(co‐IP)/wash buffer. Precipitated proteins attached on the beads were diluted in 2× SDS sample loading buffer, boiled for 10 minutes and underwent Western blot analysis.

### 2.9 Real‐Time PCR (RT‐PCR)

Eastep® Super Total RNA Extraction Kit (Promega catalog no. LS1040) was used for total RNA extraction from cell samples. RNA (1 μg) was reverse‐transcribed using a FastQuant RT Kit (TIANGEN catalog no. KR106‐02) followed by a heating (95°C, 3min) to inactivate the polymerase. The resulting cDNA was used for RT‐PCR using the following primers (5′‐3′):

USP11‐F: AGGTGTCAGGTCGCATTGAG;

USP11‐R: TGAGAGCCGGTACATCAGGA;

GAPDH‐F: AAGGTGAAGGTCGGAGTCAA;

GAPDH‐R: AATGAAGGGGTCATTGATGG;

NONO‐F: TTCCTCCCGACATCACTGAG;

NONO‐R: ATCCACAATGACTACAGCCCT

### 2.10 RNA interference and lentivirus transduction

The sequences of the USP11 siRNAs have been previously reported^7^, ^32^ (siUSP11#1:5′‐AATGAGAATCAGATCGAGTCC‐3′; siUSP11#2:5′‐AAGGCAGCCTATGTCCTCTTC‐3′). USP11 siRNAs and the control siRNA were synthesized by Shanghai GenePharma. siRNA transfection was performed as per the manufacturer's protocol (SignaGen Laboratories, Cat. SL100568). After 48 hours, the cells were harvested for the downstream experiments. For stable knockdown of endogenous USP11 expression, lentiviral shRNAs were used to infect the cells for 24‐48 hours as per the user's manual. The following shRNA target sequences (5’‐3’) were used^7^:

USP11#1, CCGTGATGATATCTTCGTCTA;

USP11#2, AAGGCAGCCTATGTCCTCTTC;

and control sequence, TTCTCCGAACGTGTCACGT.

All shRNAs were purchased from GenePharma.

### 2.11 In Vivo Ubiquitination Assay

The USP11 low‐expression SK‐Mel‐28, A375 and HEK293 stable cells, or the cells transfected with designated plasmids for 24 hours, were maintained in a medium consisting of DMEM and 10% FBS and 20 μmol/L MG132 for 6 hours. Ubiquitination assay in vivo was described in detail previously.^33^ Anti‐NONO or anti‐Myc antibodies were used to immunoprecipitate the total proteins.

### 2.12 In vivo tumorigenesis study

SK‐Mel‐28 stable cells expressing USP11 in normal/low level were selected using puromycin, and then seeded into wells of a 6‐plate at an optimized density of 3 × 10^5^ cells/well, then NONO was stably transfected into indicated cells for another 24 hours, stable cell strains with transfected shRNAs and/or vectors were generated. To obtain melanoma xenografts in nude mice, a total of 8 × 10^6^ SK‐Mel‐28 stable cells expressing the indicated shRNA and/or constructs were harvested, washed twice with DPBS, re‐suspended in 100 μL of DPBS, and injected into each 4‐week‐old specific pathogen‐free BALB/c nude mouse (n = 5 per group) on the right flank. The tumour size was monitored every 2 days for 37 days using a caliper (except the first 6 days), and the tumour volume (*V*) equals 0.5 (length × width^2^), where *L* is the longest diameter and *W* is the shortest diameter. 37 days later, the mice were sacrificed and the tumour formation rates were analysed.

### 2.13 Quantification and statistical analysis

Differences in p‐values less than 0.05 (*P* ≤ .05) were considered statistically significant for statistical analyses throughout the experiments. We reported data from at least three biologically independent experiments with similar results. GraphPad Prism version 8.0 was used in the analysis of data collected.

## 3 RESULTS

### 3.1 USP11 interacts with NONO

**FIGURE 1:**
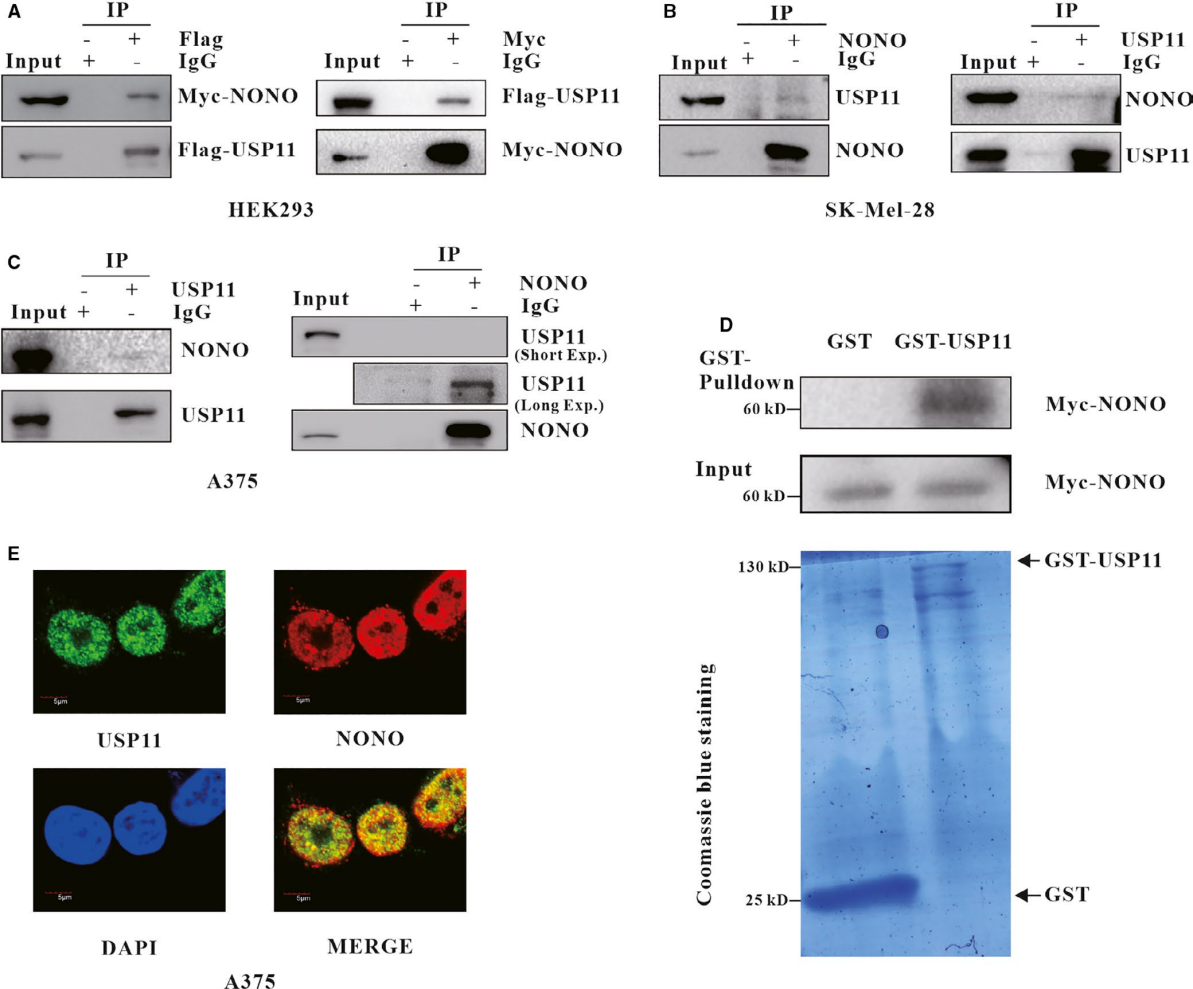
USP11 interacts with NONO. A, Flag‐USP11 plasmid and Myc‐NONO plasmid were introduced into HEK293. Antibodies against Flag or Myc, an isotype‐matched normal IgG immuno‐precipitated total cell lysates, then the indicated bands were checked in the precipitations. B, SK‐Mel‐28 cell, (C) A375 cell lysates were immuno‐precipitated with control IgG, anti‐USP11/anti‐NONO antibody. The indicated proteins were checked in the precipitations. To detect the IPed protein, the prey proteins’ bands were visualized in long exposure. D, Myc‐NONO was expressed in HEK293 cell, GST and GST‐USP11 purified from E.coli were incubated with equal Myc‐NONO, and then loaded onto GST‐tag Purification Resin, Myc‐NONO in the elution was analysed. E, The subcellular localization of USP11 (green) and NONO (red) in A375 was visualized. DNA was counterstained with DAPI, and the views of USP11 and NONO were merged

Previous studies have demonstrated that there is the possibility that USP11 and NONO may interact with each other.^34^ In order to ascertain the hypothesis, we transfected Flag‐USP11 and Myc‐NONO into HEK293 cells, collected total proteins and used the antibodies against Flag/Myc and the isotype‐matched control IgGs to perform coimmunoprecipitation (co‐IP). The results showed that Myc‐NONO was found in Flag‐USP11 precipitations but not present in IgG, and vice versa (Figure 1A). Furthermore, we investigated the interaction of endogenous USP11 and NONO in melanoma cells SK‐Mel‐28 and A375 using co‐IP. The results showed that USP11 or NONO was found in the respective precipitations but not in the normal IgG (Figure 1B and 1C). To analyse whether USP11 and NONO interacts with each other directly, purified recombinant USP11 protein was generated and GST‐pulldown assays results showed that recombinant GST‐USP11, but not the GST control, could bind to Myc‐NONO protein expressed in HEK293 cells (Figure 1D). Immunofluorescent staining assay revealed that both USP11 and NONO were co‐located in the nucleus (Figure 1E). Taken together, these results suggest there is a direct interaction between USP11 and NONO in vivo.

### 3.2 USP11 affects NONO levels

**FIGURE 2:**
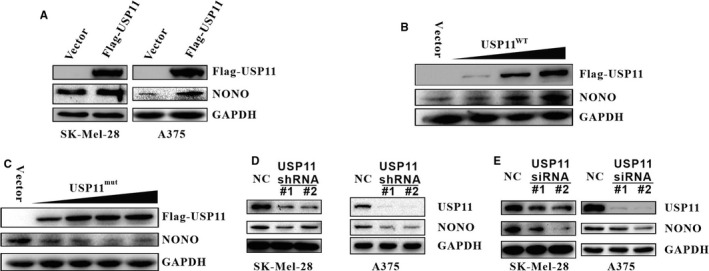
USP11 affects NONO levels. A, SK‐Mel28 and A375 were transfected with Flag‐USP11 or control vector, and the indicated proteins were assessed. B, C, A375 cells were transfected with increasing amounts of USP11^WT^ (B) or USP11^Mut^ (C275S/C283S) vector (C), total proteins were extracted and the indicated proteins were checked. D, SK‐Mel‐28 and A375 were infected with USP11‐specific shRNA #1 or #2, and the indicated proteins were analysed. E, SK‐Mel28 and A375 were introduced with either scrambled or USP11 siRNA, the indicated proteins were probed

Based upon the observed interaction between NONO and USP11 described above, we speculate that USP11 may affect NONO levels in melanoma cells. To test this speculation, USP11 was introduced into SK‐Mel28 and A375. We found that the overexpression of USP11 resulted in an increase of endogenous NONO levels (Figure 2A). Moreover, a gradual increase of USP11 expression resulted in a dose‐dependent elevation of NONO (Figure 2B). In contrast, the catalytically inactive USP11 abnormality (C275S/C283S) (USP11^mut^) could not affect NONO levels (Figure 2C), suggesting USP11 ‐regulated NONO levels in a manner that is dependent on its deubiquitination activity. Furthermore, we conducted a loss‐of‐function analysis through the use of two USP11 shRNAs in SK‐MEL‐28 and A375, the USP11 low‐expression stable cells had lower NONO level than normal cells (Figure 2D). Two USP11 siRNAs got the same results as USP11 shRNAs (Figure 2E).

### 3.3 USP11 controls NONO protein level in a proteasome‐dependent manner

**FIGURE 3:**
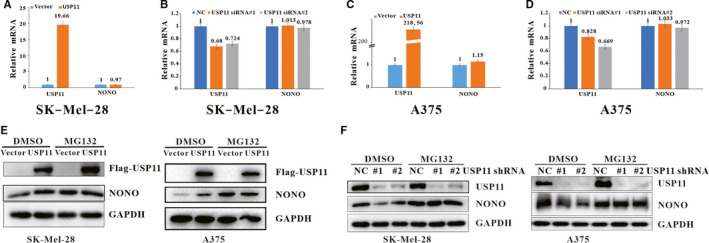
USP11 regulates NONO protein levels in a proteasome‐dependent manner. A, B, C, D, SK‐Mel‐28 cells (A and B), A375 cells (C and D) were introduced with Flag‐USP11/ control vector (A and C) or control scrambled siRNA/USP11 siRNA (B and D) , total RNA was subjected to RT‐PCR. E, F, SK‐Mel28 or A375 cells transfected with Flag‐USP11/control vector(E) or infected with indicated lentivirus shRNA (F) were treated with DMSO or MG132 (20 µmol/L) for 6 h, and the indicated proteins were probed

To explore the conceivable effect of USP11 on the mediation of NONO levels at the transcriptional level, the relative mRNA levels of NONO were assessed using RT‐PCR. Neither USP11 overexpression nor depletion in SK‐Mel‐28 and A375 cells had significant influence on NONO mRNA levels (Figure 3A, 3B, 3C and 3D), indicating that USP11 positively regulates NONO at the protein levels, but not at the transcriptional levels. To elucidate the underlying mechanism by which USP11 regulates the protein levels of NONO, Flag‐USP11 plasmid or control vector was introduced into SK‐Mel‐28 and A375, after 24 h, MG132 was added into the medium to inhibit the proteasome activity followed by the NONO examination. As anticipated, overexpression of USP11 could up‐regulate NONO in the absence of MG132, whereas MG132 pretreatment effectively eliminated USP11‐mediated change of NONO levels (Figure 3E). Comparable results were yielded after knockdown of USP11 using two specific shRNAs (Figure 3F) in SK‐Mel‐28 and A375 cells. Collectively, these results reveal that USP11 regulates NONO protein level by mediating the proteasomal degradation.

### 3.4 USP11 deubiquitinates NONO

**FIGURE 4:**
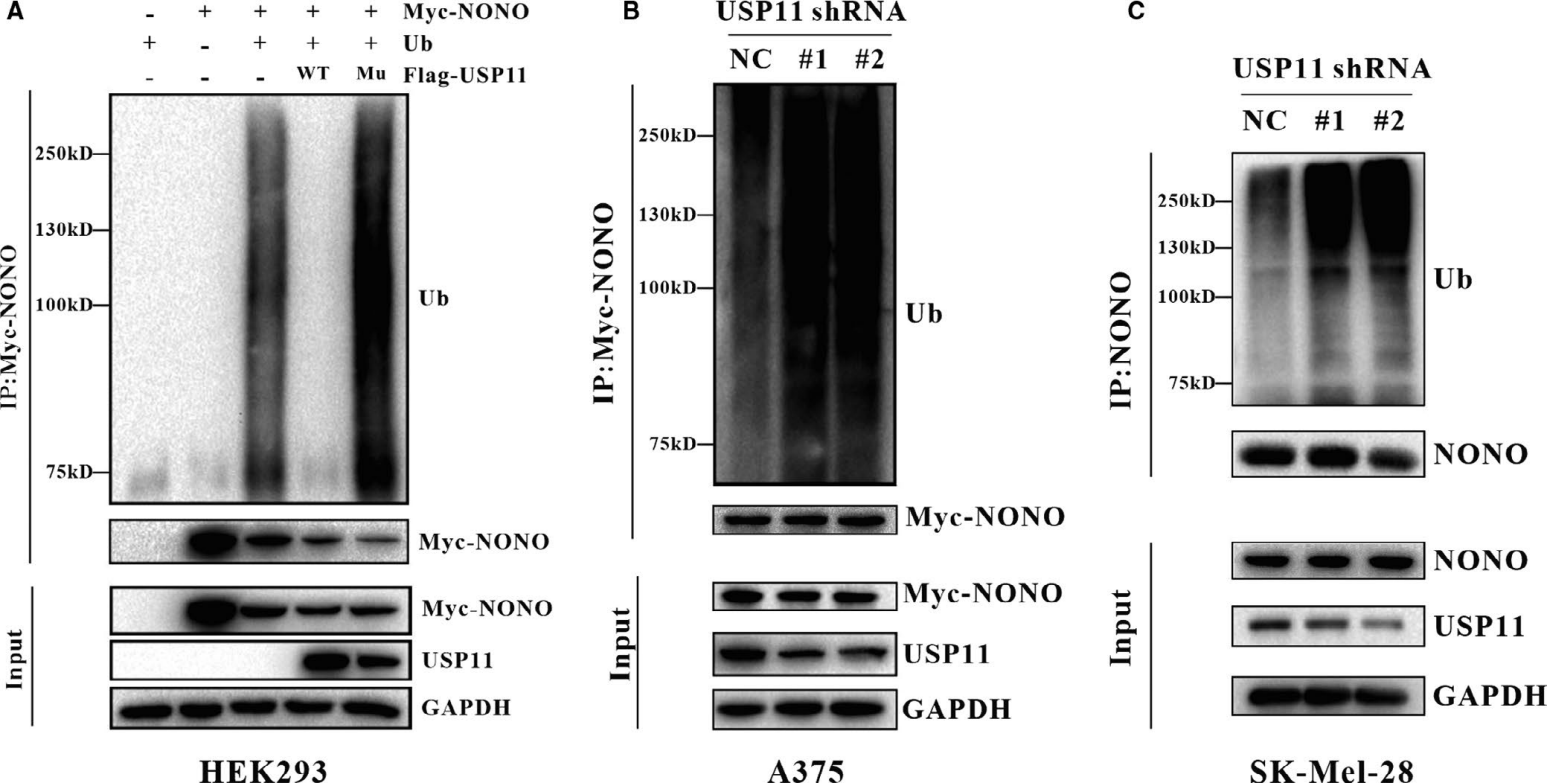
USP11 maintains NONO levels by Deubiquitination. A, HEK293 cells were transfected with the indicated vectors and then treated with MG132 (20 µM) for 6 h followed by the harvest. Myc‐NONO fusion protein was immuno‐precipitated with a Myc‐specific antibody, and the precipitations were checked with the antibody against Ub or Myc. (*WT: Wild‐Type Flag‐USP11, *Mu: Mutated Flag‐USP11 (C275S/C283S)) (B) A375 cells infected with USP11 shRNA or scrambled control shRNA, were transfected with Myc‐NONO and ub vectors, after 24 h later, the cells were treated with MG132 (20 µM) for 6 h followed by the harvest. Myc‐NONO fusion protein was immuno‐precipitated with anti‐Myc antibody, and the precipitations were checked with the antibody against Ub or Myc. C, SK‐Mel‐28 cells infected with the USP11 shRNA or scrambled shRNA were treated with MG132 (20µM) for 6 h followed by the harvest. Endogenous NONO protein was immuno‐precipitated with anti‐NONO antibody, and the precipitations were checked with the antibody against Ub or NONO

Given that ubiquitination is critical for proteasome‐mediated destruction of NONO, we hypothesized that USP11 might affect NONO ubiquitination. To test this hypothesis, we introduced an empty vector, a Flag‐tagged WT‐USP11 plasmid or mutant USP11 plasmid into HEK293 and measured poly‐ubiquitinated NONO levels. Overexpression of WT‐USP11, but not mutant USP11, reduced NONO ubiquitination (Figure 4A). Conversely, USP11 silencing with two independent shRNAs increased endogenous NONO poly‐ubiquitination in A375 and SK‐Mel28 (Figure 4B and 4C). Collectively, these results indicate that USP11 regulates NONO levels by deubiquitination and the enzymatic activity of USP11 is critical for NONO deubiquitination.

### 3.5 USP11 promotes melanoma cell proliferation via NONO

**FIGURE 5:**
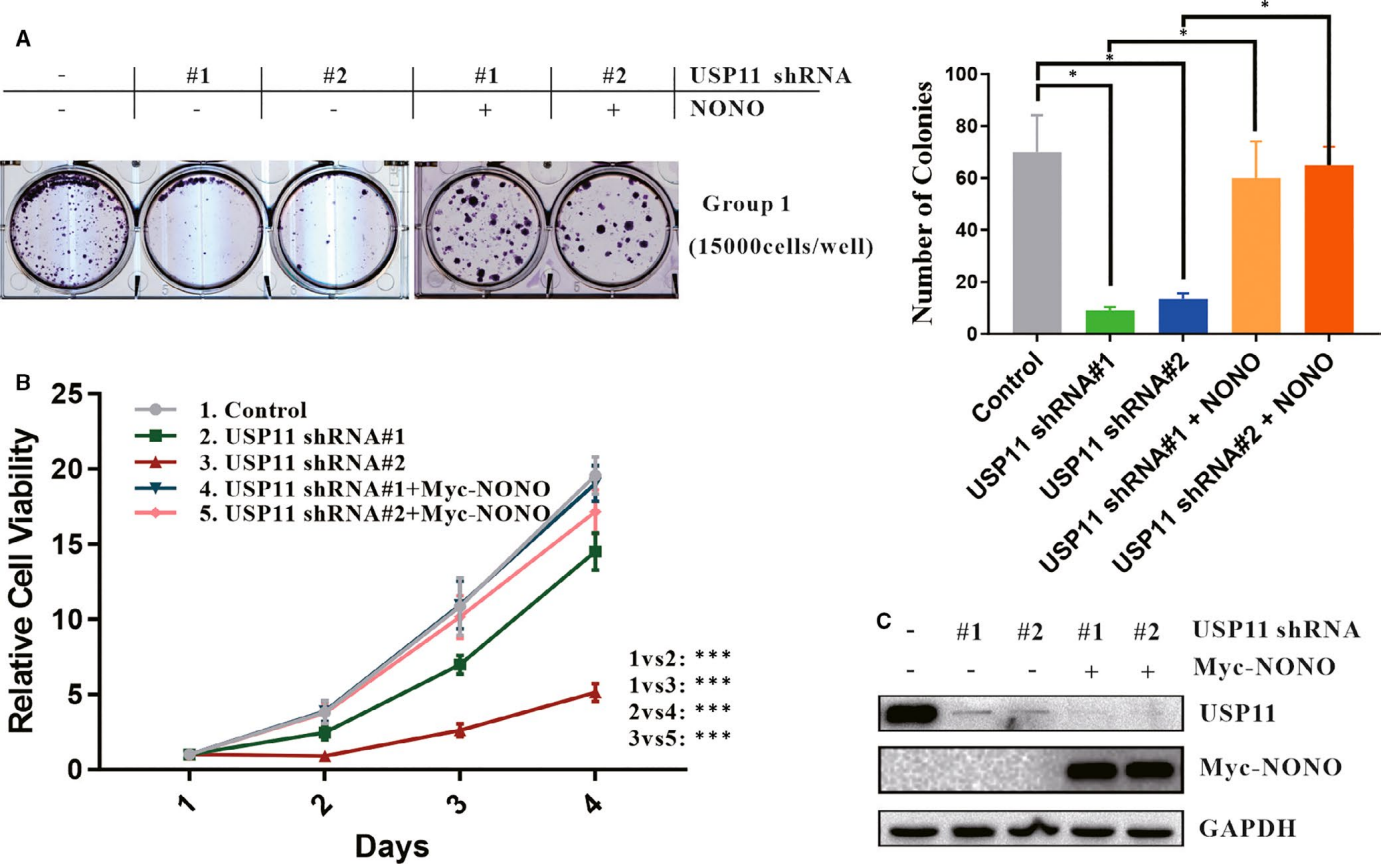
USP11 promotes melanoma cell proliferation via NONO. A, Colony formation assay was performed. A375 cells infected with lentivirus USP11 shRNA and transfected with the indicated vectors, were seeded with density 15 000 cells per well. 24 h later, A375 cells were subcultured and selected using G418 (200 µg/mL), and surviving colonies were counted 2 weeks later. Colonies were visualized and quantified. Relative cell viability was summarized from three independent experiments and was presented on the right. Statistical significance was determined by a two‐tailed, unpaired Student's *t* test. Data represent the mean (±SD) of three independent experiments (*P ≤ 0.05). B, A375 was infected with USP11‐specific shRNA #1 or #2 and then introduced with Myc‐NONO plasmid. CCK‐8 assays were performed to check relative cell viability at the indicated time points. C, A375 cells used in colony formation assay and CCK‐8 assays were lysed and analysed using Western blotting

USP11 is frequently overexpressed in melanoma and is linked to the proliferation of melanoma cells.^28^ To determine whether USP11 affects cell proliferation by acting on NONO, colony formation was performed. In congruence with previous report,^11^ USP11 knockdown inhibited the proliferation of A375, which could be reversed by the introduction of ectopic NONO (Figure 5A). Similar results were yielded from a CCK‐8 assay (Figure 5B), indicating that USP11 mediated the proliferation of melanoma cells through NONO. In addition, the effect of USP11 knockdown and reintroducing NONO expression are shown in Figure 5C.

**FIGURE 6:**
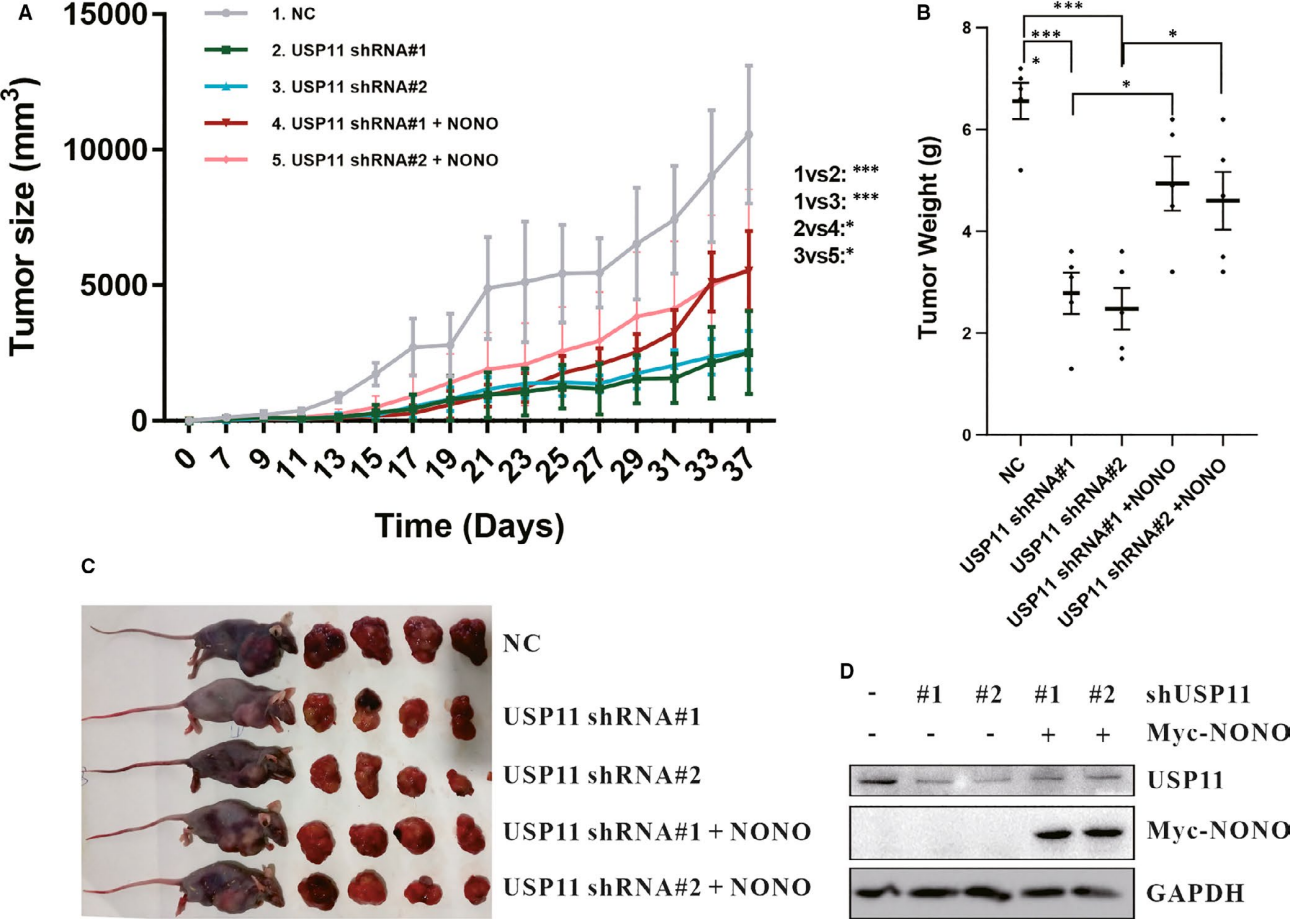
USP11 promotes melanoma progress in a NONO‐dependent manner in vivo. A, B, C, The 8 × 10^6^ indicated shRNA‐transduced SK‐Mel‐28 cells with or without ectopic expression of NONO were subcutaneously injected into mice. Tumour size (A), tumour weight (B) and tumour images (C) were shown. D, SK‐Mel‐28 cells implanted into nude mice were lysed and analysed using Western blotting

To determine the oncogenic function of USP11 in melanoma in vivo, xenograft experiments were performed using USP11‐depleted SK‐Mel‐28 cells which were inoculated into nude mice. Tumour growth was measured at defined time intervals. Compared with mice bearing control‐shRNA‐transfected cells, mice implanted USP11‐shRNA–cells indicated reduced tumour growth throughout the experiment. At the 37‐day mark after inoculation, the volume and weight of the tumour formed by USP11‐depleted SK‐Mel‐28 cells significantly decreased. Nevertheless, restoring NONO expression reversed the tumour‐suppressing effect of USP11 shRNAs (Figure 6A, 6B and 6C). Analysis via Western blot verified that the effects of USP11 knockdown and reintroducing NONO expression were retained in these tumours (Figure 6D). Collectively, our data showed that USP11 has a NONO‐dependent tumour‐promoting function.

### 3.6 USP11 is overexpressed and positively correlates with NONO in melanoma

**FIGURE 7:**
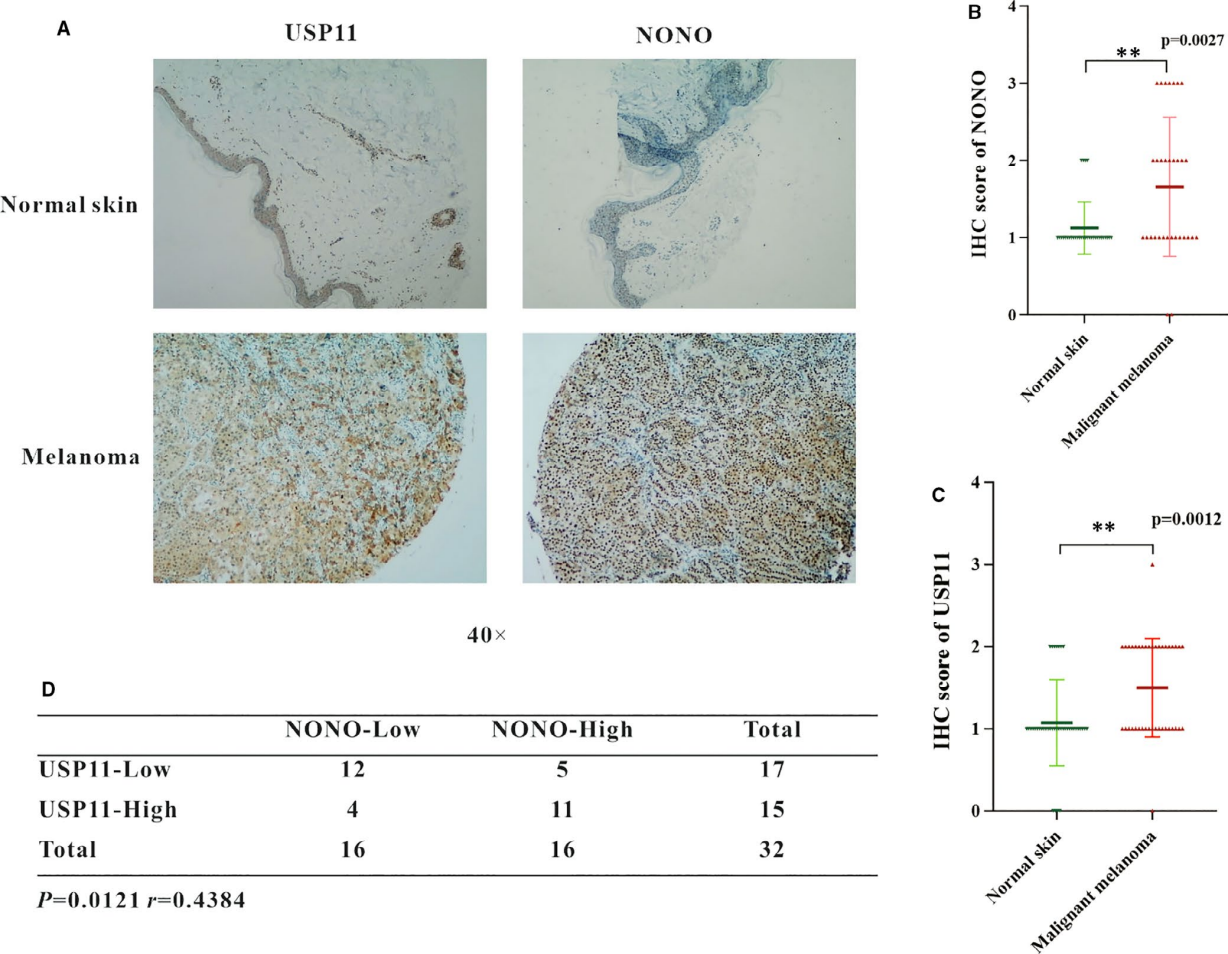
USP11 is overexpressed and positively correlates with NONO in melanoma. A, Representative images of USP11 and NONO protein in normal skin tissue and melanoma skin tissue. B, C, NONO (B) and USP11 (C) protein levels in normal skin and melanoma skin tissues. D, Correlation study between USP11 and NONO in clinical melanoma skin tissues. The statistical analysis was determined by a χ^2^ test

To investigate the relevance of USP11 and NONO abundance in human melanoma, an immunohistochemical analysis was performed to determine the protein levels of USP11 and NONO in 64 specimens including 32 normal skin tissues and 32 melanoma tissues. Representative stains of USP11 and NONO in normal skin tissue and melanoma tissue are shown in Figure 7A. In melanoma tissues, high expression of USP11 and NONO was observed in 15 (46.88%) and 16 cases (50%) respectively, whereas only 8 (25%) and 4 (12.5%) cases respectively in normal skin tissues (Figure 7B, 7C). This suggests that both USP11 and NONO are overexpressed in melanoma. Moreover, there was a significant positive correlation (*R* = 0.4384, *P* = 0.0121) between USP11 and NONO in those melanoma tissues (Figure 7D). These results suggest that expression of NONO is correlated with USP11 and USP11 may stabilize NONO protein to enhance tumorigenesis in melanoma patients.

## 4 DISCUSSION

In this study, we identified USP11 as the first deubiquitinase for NONO. We demonstrated that USP11 interacted with NONO, removed poly‐ubiquitin chains conjugated onto NONO. As a result, exogenous USP11 increased NONO levels by preventing its ubiquitination. By contrast, knockdown of USP11 decreased NONO levels, which was accompanied by increased ubiquitination. Thus, our results suggest that NONO levels were dynamically regulated at the post‐translational level by ubiquitination and deubiquitination.

Recently, two E3 ubiquitin ligases have been identified to promote NONO ubiquitination and degradation. One such ligase is FBW7, a SCF(SKP1/CUL1/F‐box)‐type ubiquitin ligase which mediates NONO ubiquitination involved in GSK3β kinase modulation.^29^, ^35^ The other ligase is RNF8, which can promote NONO degradation to switch off signalling through the ATR‐CHK1 pathway following UV‐induced DNA damage.^30^ Three lysine residues of NONO (K278, K290 and K295), out of a total of 27, are essential for UV‐induced RNF8‐dependent NONO degradation.^30^ Here, we further revealed that NONO ubiquitination could be reversed by USP11 to avoid its degradation through the ubiquitin‐proteasome pathway. Interestingly, previous studies indicate that USP11 and RNF8/RNF168 function together to regulate the γH2AX levels by deubiquitination and ubiquitination.^36^ Although we don't provide evidence in this study to show whether USP11 could resist the effects of FBW7 or/and RNF8 on NONO, we speculated that the cellular levels of NONO may depend on the fine regulation among USP11, FBW7 and RNF8. Additional studies are warranted in order to fully understand the regulation mechanism of NONO levels.

USP11 belongs to the USP family of deubiquitinases and has been reported to stabilize multiple cellular proteins by reversing their ubiquitination, such as PML,^37^ p21,^7^ E2F1,^38^ ARID1A^8^ and XIAP.^39^ In this study, we further demonstrate that NONO is a novel substrate of USP11. The cellular NONO levels can be regulated by deubiquitination mediated by USP11. However, what is surprising is that we failed to observe the impact of USP11 on the stability of NONO in cells treated with the protein synthesis inhibitor cycloheximide (CHX). The reason may be that NONO is a protein with a long half‐life. It is reported that the half‐life of NONO is about 32 hours in HeLa cells, which is consistent with in silico prediction.^29^ As a result, the cells need to be treated with CHX for a lengthy amount of time, subsequently causing serious cytotoxicity.^29^

The dysregulation of USP11 had been found in a variety of human cancers. Interestingly, USP11 in different cancers exhibits distinct roles. USP11 serves as a tumour suppressor in lung cancer,^7^, ^40^ brain tumours^37^ and squamous cell carcinomas^8^ but has a tumour‐promoting role in colon cancer,^41^, ^42^ cervical cancer^43^ and breast cancer.^39^, ^44^ The dual behaviour of USP11 may depend on its regulated substrate proteins. In melanoma, overexpression of USP11 has been frequently observed and is correlated with poor prognosis.^11^ In congruence with this, our results indicate that USP11 acts as an oncogene, because USP11 overexpression promotes the proliferation of melanoma cells, whereas knockdown of USP11 exhibits the opposite function. Furthermore, we demonstrate that USP11 mediates the proliferation of melanoma cells via NONO because the effect of USP11 knockdown on melanoma cells could be rescued by introducing NONO. Moreover, a significant positive correlation between USP11 and NONO abundances were found in clinical melanoma samples. Thus, our study shows that USP11‐NONO signalling axis plays a critical role in melanoma proliferation. However, future studies are needed to ascertain how USP11 expression is regulated and how transcription and post‐translational modification of NONO coordinate the cellular level of NONO, which will provide clues for effective control of melanoma.

## CONFLICTS OF INTEREST

The authors confirm that there are no conflicts of interest.

## AUTHOR CONTRIBUTION

**Peifu Feng:** Data curation (lead); Formal analysis (equal); Investigation (equal); Methodology (equal); Validation (equal); Writing‐original draft (lead). **Ling Li:** Data curation (equal); Formal analysis (equal); Methodology (equal); Validation (lead); Visualization (equal). **Jing Dai:** Data curation (equal); Formal analysis (equal). **Lingli Zhou:** Data curation (equal); Formal analysis (equal); Validation (equal). **Jing Liu:** Conceptualization (equal); Writing‐review & editing (equal). **Jinfeng Zhao:** Writing‐review & editing (equal). **Xiaodong Li:** Data curation (equal); Formal analysis (equal). **Neng Ling:** Validation (equal). **Siyuan Qiu:** Validation (equal). **Lin Zhang:** Data curation (equal); Formal analysis (equal); Validation (equal). **Tiantian Xie:** Investigation (equal). **Yinglei Chen:** Investigation (equal). **Tianhuan Peng:** Visualization (equal). **Michael John Donovan:** Writing‐review & editing (equal). **Jianhui Song:** Conceptualization (equal); Project administration (equal); Supervision (equal); Visualization (equal); Writing‐review & editing (equal). **Mao Ye:** Conceptualization (lead); Funding acquisition (lead); Project administration (lead); Supervision (lead); Writing‐original draft (equal); Writing‐review & editing (equal).

## Data Availability

The data used to support the findings of this study are available from the corresponding author upon request.
